# MERGING conventional and complementary medicine in a clinic department – a theoretical model and practical recommendations

**DOI:** 10.1186/s12906-015-0696-2

**Published:** 2015-06-09

**Authors:** Marion Pérard, Nadine Mittring, David Schweiger, Christopher Kummer, Claudia M. Witt

**Affiliations:** Institute for Social Medicine, Epidemiology and Health Economics, Charité - Universitätsmedizin, Berlin, Germany; Institute for Complementary and Integrative Medicine, University of Zurich and University Hospital Zurich, Sonneggstr. 6, CH-8091 Zurich, Switzerland; Schweiger & Associates, Hilton Head Island, SC USA; Institute of Mergers, Acquisitions and Alliances (IMAA), Zurich, Switzerland

**Keywords:** Merger, Fusion, Complementary medicine, Health management, Corporate culture, Integrative medicine

## Abstract

**Background:**

Today, the increasing demand for complementary medicine encourages health care providers to adapt and create integrative medicine departments or services within clinics. However, because of their differing philosophies, historical development, and settings, merging the partners (conventional and complementary medicine) is often difficult.

It is necessary to understand the similarities and differences in both cultures to support a successful and sustainable integration. The aim of this project was to develop a theoretical model and practical steps that are based on theories from mergers in business to facilitate the implementation of an integrative medicine department.

**Methods:**

Based on a literature search and expert discussions, the cultures were described and model domains were developed. These were applied to two case studies to develop the final model. Furthermore, a checklist with practical steps was devised.

**Results:**

Conventional medicine and complementary medicine have developed different corporate cultures. The final model, which should help to foster integration by bridging between these cultures, is based on four overall aspects: culture, strategy, organizational tools and outcomes. Each culture is represented by three dimensions in the model: corporate philosophy (core and identity of the medicine and the clinic), patient (all characteristics of the professional team’s contact with the patient), and professional team (the characteristics of the interactions within the professional team).

**Conclusion:**

Overall, corporate culture differs between conventional and complementary medicine; when planning the implementation of an integrative medicine department, the developed model and the checklist can support better integration.

**Electronic supplementary material:**

The online version of this article (doi:10.1186/s12906-015-0696-2) contains supplementary material, which is available to authorized users.

## Background

In recent years, the use of complementary medicine has risen [1, 2]; in particular, cancer patients ask for more holistic treatments [3–7] such as acupuncture for reducing the nausea caused by chemotherapy. The broad field of complementary medicine is defined by the National Center for Complementary and Alternative Medicine (NCCAM) at the National Institutes of Health (NIH) in the US as “a group of diverse medical and health care systems, practices and products that are not generally considered to be a part of conventional medicine. Complementary medicine is used together with conventional medicine, and alternative medicine is used in place of conventional medicine” [8].

Patients’ high demand for complementary medicine therapy exerts pressure [9] on clinics to adapt appropriately to patients’ needs. In many places, this adaptation has resulted in a shift from the separation of conventional and complementary medicine to a “merger” of the two medicine fields [10]. New terms have been introduced that aim to capture the increasing integration of complementary medicine in a conventional medicine setting, particularly the term “Integrative Medicine” [11]. This term was defined by the Consortium of Academic Health Centers for Integrative Medicine as “the practice of medicine that reaffirms the importance of the relationship between practitioner and patient, focuses on the whole person, is informed by evidence, and makes use of all appropriate therapeutic approaches, healthcare professionals and disciplines to achieve optimal health and healing” [12]. In countries such as Germany, where both complementary and conventional medicine are often provided by conventional medicine physicians, integrative medicine was described as the combination of mainstream with complementary medicine, supposedly leading to synergistic therapeutic effects [13]. More patient-centered care might suggest a more integrative medicine approach that combines the best of conventional medicine with the best of complementary medicine. However, because of their differing philosophies, historical development, and settings, merging conventional and complementary medicine can be very challenging [14, 15]. It is necessary to understand the similarities and differences in both cultures to support a successful and sustainable integration.

In the business environment, when organizations merge, understanding cultural similarities and differences in these organizations—in other words, their individual corporate cultures—is a necessity. Each organization has its own identity, personality and way of conducting its business, and these specific aspects make an organization unique. Davenport (1998) describes corporate culture as “the DNA of an organization, invisible to the naked eye, but critical in shaping the character of the workplace” [16]. Corporate culture is also the “collective programming of the mind” that distinguishes the members of one organization from another [17]. Cartwright and Cooper define corporate culture simply as “the way in which things get done within an organization” [18], in other words: making people speak the same language.

With a merger, organizations have the opportunity to adapt quickly to new or changing markets by permitting the more rapid transformation of the organization than organic growth might allow [19]. In economics, a merger is defined as “the combining of two or more entities into one, through a purchase acquisition or a pooling of interests“ [20]. The careful selection of merger partners is extremely important for success. Not only must the acquirer consider the likelihood of success of combining the financial and strategic aspects of both organizations, it must also consider the likelihood of success of combining the corporate cultures [18]. Corporate culture determines individuals’ commitment, satisfaction, productivity and longevity with an organization [21] because individuals tend to select organizations with which their own values are aligned [22]. When an individual’s values fit well with the corporate culture, a psychological bond is formed and is difficult to break [23].

It is widely recognized that cultural differences between merger partners are one of the most common reasons for failure [18, 24]. Any aspect of disagreement may be a point of failure (e.g., communication problems within the team, high turnover) [25].

A prominent example is the merger of Daimler-Benz with Chrysler. This merger seemed to make sense from a business perspective, but the contrasting cultures have impeded the development of positive synergies [24]. Daimler-Benz honors traditional hierarchy and methodical decision-making, whereas Chrysler stands for pragmatic adaptability, creativity and equal empowerment [26]. In general, two merging organizations need not necessarily have similar or the same corporate cultures, but they should be able to act together. Therefore, two aspects are important: the degree to which the cultures are different and in which direction the cultural change should proceed [18]. If the change proceeds in the direction of increasing individual freedom, the integration may be easier because the new culture might seem to be more appealing than the previous one [18]. In addition, the willingness of an employee to abandon his/her culture depends simultaneously on the consideration of that culture and on the attractiveness of the other [18].

Approaches for the degree and depth of combining two companies in a merger can vary. The “confederation” approach combines organizations that work in parallel with no integration. In the “linking” type, the organizations work together with no real integration. The “absorption” type is when the acquired organization is fully absorbed by and becomes a part of the acquirer. The acquired organization has to fully adopt the corporate culture of the acquirer; there is no creation of a new corporate culture. The first approach, in which the creation of a new corporate culture is needed, combines the advantages of both organizations in the “best of both worlds” method. The integration level is high, and therefore, a strong new corporate culture is needed to bind the two groups together [27].

Overall, the framework of corporate culture in business mergers seems to be suitable for applying to the mergers of conventional and complementary medicine into a new entity labeled “integrative medicine.” The aim of this project was to develop a theoretical model and practical steps that are based on business merger theories to facilitate the implementation of an integrative medicine department.

## Methods

We conducted a literature search on cultures in conventional and complementary medicine. We searched Pubmed and the internet by combining the terms culture, philosophy, work style, work manner, patient-practitioner relationship, time devoted to the patient with integrative medicine, complementary medicine, alternative medicine and CAM. Furthermore, we asked medical anthropologists for additional literature that is not available in Pubmed (e.g., books). The literature for the narrative review was analyzed with a focus on extracting information on various aspects of culture, such as the philosophy, work style, and characteristics of the physician/practitioner-patient relationships in conventional and complementary medicine. The results of the literature search on culture in conventional and complementary medicine were successively condensed and are summarized in the results section and in Table 1. We also conducted a literature search on merger and corporate culture theories using the terms role of corporate culture in merger, corporate culture in merger, professional culture, reasons for merger failures, merger of medical traditions, and fusion or merger of conventional and complementary medicine. From that search, only the model “15 behavioral dimensions of organizational culture” from the Schweiger-Larkey-Group, known as SLOCI, was identified [28]. We contacted Schweiger as well as Kummer as international well-known experts in the field and invited them to participate in this project. According to their knowledge, no other models relevant to our research aim have been published. The SLOCI dimensions are targeted to highlight essential key differences in the corporate cultures between two merger partners, which can lead to substantial clashes. Therefore, this model is suitable for establishing cultural prerequisites for a merger. However, as the literature analysis on the cultures of conventional and complementary medicine revealed, additional aspects need to be taken into account, for example, the medical philosophy, physician/practitioner-patient interactions and medical expertise because mergers occur on different levels (patient, professional team, clinic, institution, regulation, system) [10, 29]. To keep the model simple, appropriate, and manageable, we did not want to go into too much detail for each aspect, and we defined the dimensions more broadly than did the SLOCI. Nevertheless, the 15 SLOCI dimensions can be incorporated into our model. For example, “cautious communications versus open communications” [28], “deliberate communications versus fast communications” [28] and “indirect communications versus direct communications” [28] can be found on one hand under “professional team – communication” and on the other hand under “patient – communication”.

**Table 1:** Major cultural differences in corporate philosophy between conventional and complementary medicine

	Conventional medicine	Complementary medicine
Values		
Philosophy of care	Positivistic approach [36]:	Holistic approach: Bio-psycho-spiritual-social model [35, 36, 45]
	• Importance is given to the knowledge of facts and experimental sciences [36]	• The whole is more than the sum of its parts
	• The patient is given the undivided clinical attention of the physician [52]	• Body, mind and spirit are interrelated and must all be considered in healing
		• Aims neither unilaterally at the body nor at the soul but treats the patient as a whole
Philosophy of healing	• Health: − “A state of complete physical, mental and social well-being and not merely the absence of disease or infirmity” WHO Constitution [39] = criticized definition [53] as static and accentuating only subjective aspects [39]	• Health, disease and therapy effects do not result solely from molecular interactions but also from the different causal interactions between these factors within the human being as a whole. [45]
	- Other definitions are “ex-negativo” explanation: [54]	• Healing = (re)establishment of the harmony between the functions of body, soul and spirit [45]
	= Lack of deviance from biological norms [39], “Life with organ’s silence” [54]	• Disease = disequilibrium between biological, psychological, social and spiritual forces [55]
	• Disease = deviance from biological norms [39]	
Norms - Therapeutic approach		
Disease-oriented [44]		Patient-oriented [44]
Specialization:		Holistic approach [32, 34, 35]
• Opportunity for high competency in specialty fields [34, 39]; more efficiency [40]		• Patients’ involvement, empowerment and responsibility in the self-management of their illnesses [32, 34, 36, 42, 45]
• Routine [40, 43]		• Self-regulation of the body and its healing power; enhancing natural body reactions [34]
• Fragmentation of care (with communication and cooperation impediments) [34, 39]		• Symptoms seen as a message from the organism, similar to an SOS [35]; look at underlying causes [45]
• Risk of losing the overall vision [34]		
• Analytical [32, 34, 35]		• Intuitive [32, 34]
• Deductive [32]		• Inductive [32]
• Standardized [40]		• Tailored to individual needs [32, 44, 45]
• Evidence-based [37]; scientific [32, 34, 35]		• More or less spiritual therapeutic approaches [38]
Use of pharmacotherapy with predominantly proved effects [38] and high use of technology [43, 54]		Use of natural treatments and remedies [45] with less technical equipment than CM [45]
Focus more on structure than outcomes:		Focus more on outcomes than structure:
The quality of structure includes the personal, spatial, temporal, technical and organizational conditions of medical practice: availability, short waiting times, training and education [36]		Outcome quality refers to therapeutic goals, such as improving and healing, patient satisfaction and quality of life, encouraging health-related behavior and self-responsibility, stimulating self-regulation, prevention [36]

To test the completeness and feasibility of our preliminary model, we performed two case studies in integrative oncology centers: one in Germany (11 interviews) and one in the US (9 interviews). The results of the case studies on corporate culture in clinics were reported in a separate manuscript [30]. Both case studies consisted of interviews with different professionals (from conventional medicine, complementary medicine and administration) in each clinic, focusing on their corporate cultures. The interview guidelines for the first case study (Germany) were based on the preliminary model. The results from the interviews were used to revise our model, write the interview guidelines for the second case study (USA), and create the first version of the checklist based on the model and on the integration process described by Cartwright and Cooper [18]. After the second case study, the model and the checklist were again revised and presented at a consensus workshop to merger experts and integrative oncology experts. Comments from this workshop were included in the final model, and recommendations for general strategic dimensions and for overcoming cultural differences were educed [31].

## Results

First, we will summarize the cultural aspects of conventional and complementary medicine that were identified from the literature analysis. Subsequently, we will introduce the model and the checklist.

### The culture of conventional medicine

In the existing literature, the philosophy of conventional medicine has been described as scientific [6, 32, 33], analytic [34, 35] and deductive [32], and the data should be measurable [35] (see Table 1). With this pharmaceutically, evidence-based, and pathogenically oriented model [6, 36–38], importance is given to the “knowledge of facts and experimental sciences” such as a “rationalistic view of therapeutic modalities” [36]. Technology is an important tool in arriving at a diagnosis. Its expedience supports conventional physicians’ capacity to make quick, accurate diagnoses, decisions and treatment recommendations that result in patients’ positive outcomes, especially within pressured timeframes. The healing approach is presented as reductionist [10, 35], and, since 1945, conventional medicine has become increasingly specialized [39]. This specialization provides patients with the opportunity to be treated more efficiently [40] by highly competent clinicians in the special field they need [39]. The generalizable and standardizable [10, 32, 40] nature of the therapy is essential for conventional medicine. In essence, hospitals are comparable with organizations, with costs, revenues, staff, suppliers, clients and competition; therefore, productivity plays a key role in clinics. Clinicians are also responsible for improving financial performance and organizational efficiency and quality [41]. The role of physicians is becoming more administrative; one-third of their work is consumed with such responsibilities [40, 42]. Physicians are now service providers [40], and their tasks must be standardized, preplanned and routine-oriented [43] in order to achieve the highest efficiency. The treatment of individual cases generally conforms to a well-established therapeutic framework [44].

### The culture of complementary medicine

In the literature, the philosophy of complementary medicine is described as holistic [10, 32, 45], empowering [32, 42, 45], individualistic [32], inductive [32, 34] and intuitive [32]. Holism postulates that the whole is more than the sum of its parts [35, 36]. Supporting the body [34] and the whole person in an effort to create or reestablish balance and harmony [36] in a patient’s bio-psycho-socio-spiritual aspects [32, 36] plays an important role (see Table 1). Complementary medicine is seen to stimulate the healing power of the organism [34, 35], and symptoms are often regarded as signals of the patient’s condition, the therapy and its effects [35]. Patients are seen as unique, and therapy is individualized accordingly [35]. In interactions, the practitioner needs to communicate with the patient in a 'calm' and 'unrushed' manner [45], a practice that generally requires more time than in interactions with a conventional medicine physician [42, 45–47]. In complementary medicine, the patient is the center of the medical process [45]. In-depth conversations [42, 45] characterized by the physician’s relational and supportive communication style [45] are used to strengthen the patient-physician relationship [42, 45]. This interaction style is seen to empower the patient to take responsibility for his/her healing and therapy [34, 42, 45] and encourages shared decision-making [42, 45].

### Model and checklist

**Fig. 1:**
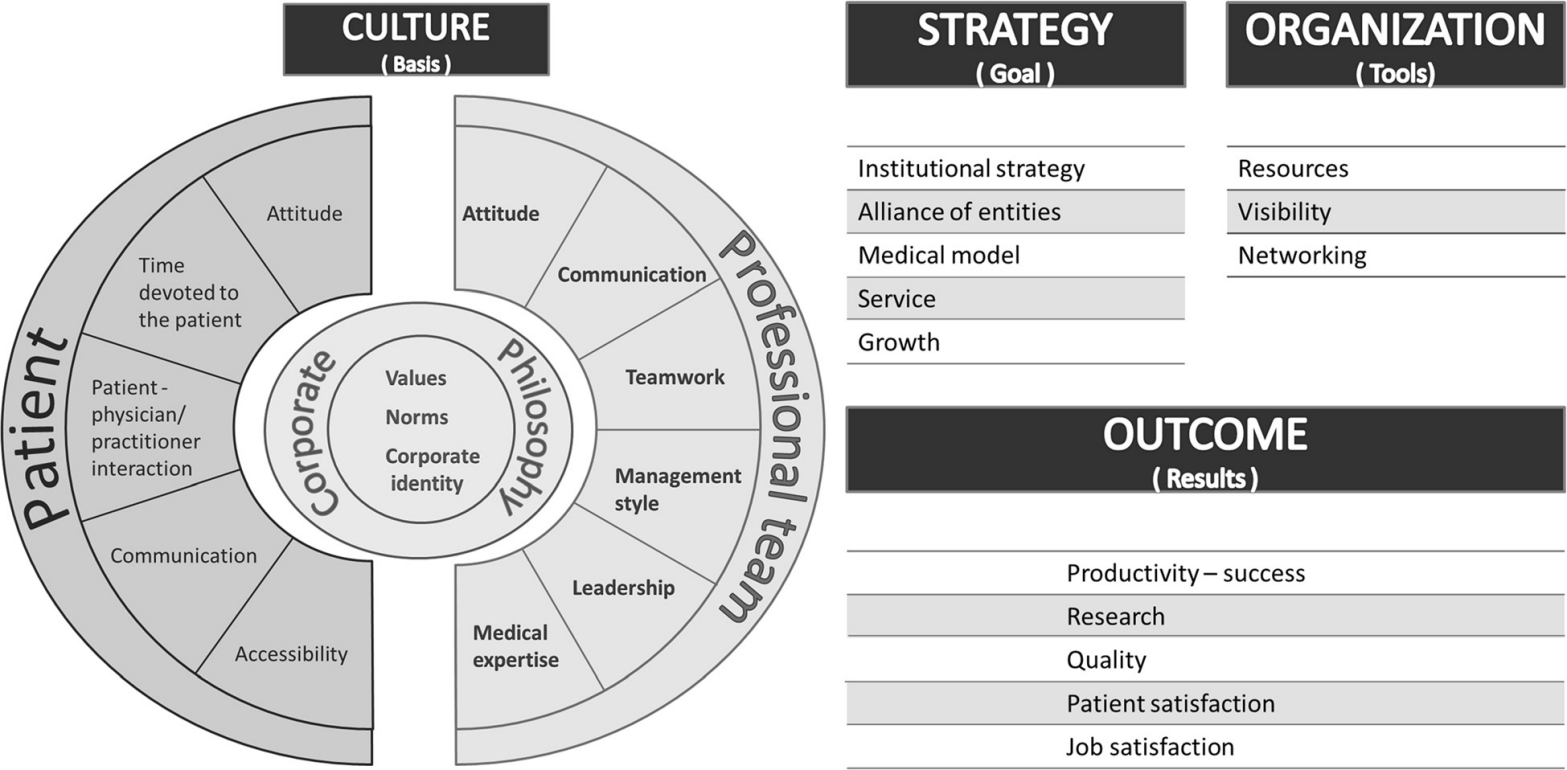
Theoretical model of the key aspects of a merger. The model is based on four overall aspects: culture, strategy, organizational tools and outcomes, with a primary focus on culture. The model should help to foster the integration of conventional and complementary medicine by bridging between the two cultures. Source: Schweiger Larkey Group: The SLOCI 15 Dimensions [http://www.sloci.com/sloci/sloci-dimensions.htm]

The final model is based on four overall aspects: culture, strategy, organizational tools and outcomes [see Fig. 1], and all of these dimensions are defined in Additional file 1: Table S1.

Each culture is represented by three dimensions in the model:

- Corporate philosophy (core and identity of the medicine and the clinic)
- Patient (all characteristics of the professional team’s contact with the patient)
- Professional team (the characteristics of the interactions within the professional team)

The main focus of the model is on culture. Nevertheless, the aspects of strategy, organizational tools and outcomes play relevant roles in the integration process. There is a need to define clear goals in the form of a strategy that includes concepts that reflect the medical model and the provided services, as well as a need to clearly define how the alliance of conventional and complementary medicine should appear. In the first two to three years, merger strategy should focus on long-term investments without expectations for profit making. To implement the strategy, organizational tools, and, especially, available resources, should be clarified. Human, financial and material resources are to be considered and should be accessible and substantial. Furthermore, the outcomes must be defined and measured. The outcome “research” is a key point for the acceptance of integrative medicine.

Based on the merger theory of Cartwright and Cooper (2009) and our final model, we developed a checklist of the sequential steps that are necessary for a successful and sustainable integration process, which should be kept as short as possible [see Additional file 2: Table S2]. The first stage is “courtship”. The management team investigates the status of complementary and conventional medicine and shareholders’ motivations to integrate complementary medicine. The strategy should be planned at this stage; the culture differences between complementary and conventional medicine should be revealed, a new corporate identity should be created, and the appropriate staff should be chosen. The second stage is the “legal announcement of the marriage”. This announcement should trigger a wave of communication with all shareholders about the merger and the new corporate culture. This stage is decisive; the employees have to know what is going on to create initial enthusiasm and synergies and to avoid the stress of uncertainty, which can lead to turnover. After this comes the “honeymoon period”. This is the moment of actual confrontation between the two worlds. The integration team implements the new corporate culture and continues to over-communicate the goals of the merger. The new corporate culture begins to take root. In the last stage, “establishing marital allegiance”, the culture is established and the integration team should attempt to maintain high visibility and remain vigilant to patient and employee dissatisfaction.

## Discussion

Using an innovative and unique approach, we developed a model that can be used to support the development of a successful and sustainable integrative medicine department in a clinic. By understanding cultural differences and creating a new, strong corporate culture aligned with the integrative medicine philosophy, teams from different backgrounds can be unified (conventional and complementary medicine). This model is accompanied by a checklist that identifies sequential steps and clarifies accountability during the process.

With the model and checklist, we bring together knowledge and experience from different fields, including business and medicine. Considering the uniqueness, novelty, and interdisciplinarity of our project, we were not able to draw upon previous results. Our project benefitted from the combination of different methodologies, including literature analyses, expert discussions, and case studies, which allowed us to control for the model’s validity and feasibility during the development process. Nevertheless, the uniqueness of the approach can also be seen as a limitation because there are no comparison models. Culture is a very broad and heterogeneous field, and we summarized the literature in a narrative review. It is possible that a systematic review would have provided a broader picture. Our model reflects this diversity and can be generally applied to different settings, including different countries with different health systems, as well as to different specialties in conventional and complementary medicine. Pragmatically, we assumed that the cultural differences between conventional and complementary medicine were fundamental factors in developing an integrative medicine department. We must also be aware that in each hospital, different kinds of cultures and subcultures already exist, including national, corporate, or professional cultures. These cultures are independent but still related.

We have chosen to compare the cultures of both complementary and conventional medicine with the cultures of two different organizations. We selected corporate culture as a starting point because it, like conventional and complementary medicine, can be explored as the way of doing things in the workplace regardless of one’s hierarchical position or profession. The dimensions of the model are not exhaustive, but for simplicity’s sake, we focused on the dimensions that in our case studies had posed the greatest challenges during integration. In our case studies and model, we focused on Western culture. We conducted case studies in two countries, the USA and Germany. In the non-Western world, cultural aspects may be different. Our aim was to analyze the merger of the corporate cultures in conventional and complementary medicine in order to understand the decisive levers for creating an integrative medicine service in a clinic. This kind of merger does not map perfectly to what happens in business when two organizations merge to one. Nevertheless, our focus was on the corporate culture aspect within mergers, and this fits also on a theoretical level because, as the literature revealed, differences in both cultures can make combining the two medicines difficult. In the present study, the definition of merger serves not only as a metaphor but also as a description of a social process. The teamwork between merger partners is decisive for the success of the merger and dependent upon the compatibility of each individual corporate culture with the other. Complementary and conventional medicine teams complement each other. In the literature, conventional medicine is described as reductionist [10, 35] and disease-oriented [34, 44], whereas complementary medicine is described as more holistic [36, 45] and patient-oriented [44], and even more extreme views on both conventional medicine and complementary medicine exist. In reality, the cultural aspects of both conventional and complementary medicine will vary according to the setting, the country and the profession.

Two key points of our model are communication and resources. Many different types of communication are implicated: internal (within the integrative medicine team, with the management or integration team) and external (with the patient, with other departments of the clinic, with the public). The most difficult communication will center on the merger and integration itself. Therefore, we recommend creating an integration team [48, 49] and maintaining continuous communication regarding merger goals with all stakeholders. A new shared language should be created through common efforts of both merger partners in order to avoid the Tower of Babel effect [50, 51].

In order to empower the integration team to succeed, the staff should be composed of knowledgeable, open-minded, stable, friendly, respected and respectful, committed, motivated, enthusiastic and realistic members. Such a team is able to exploit the model and the checklist to their full advantages and create a successful and sustainable combination of conventional and complementary medicine within a clinic.

## Conclusion

We have used an innovative interdisciplinary approach to contribute to more comprehensive and efficient patient care. We brought together knowledge and findings from corporate culture in business mergers, literature analyses and two case studies that we developed. In doing so, we demonstrated that there are major cultural differences between conventional and complementary medicine. To bridge these differences and to suggest strategies for perfectly integrating the best of both medicines, we finalized a theoretical model and a practical checklist. These allow for the systematic development of a sustainable integrative medicine service or clinic that combines conventional and complementary medicine at a high-quality level.

## Additional files

Additional file 1: Table S1. Merger culture project - operational definitions [56–62]. Additional file 2: Table S2. Detailed steps to successfully offer integrative medicine.
