# Supplementary material for: MERGING conventional and complementary medicine in a clinic department – a theoretical model and practical recommendations
Source: BMC Complement Altern Med. 2015 Jun 9;15:172. doi: 10.1186/s12906-015-0696-2 (PMC4459674; doi:10.1186/s12906-015-0696-2)
Supplement: Additional file 1: Table S1. — Merger culture project - operational definitions [56–62]. [file 12906_2015_696_MOESM1_ESM.doc]

**Additional file 1: Table S1: Merger culture project - operational definitions**

| **CORPORATE CULTURE**  The collective programming of the mind that distinguishes the members of one clinic service from another [17].  Example: “In the integrative medicine center, we build team spirit through team-building activities on the weekends”.  **Corporate philosophy**  The theory used to determine how the clinic is formed and how it manages different areas of operation such as accounting, management, training, public relations, marketing and business operations [56].  Example: “We want to focus on the human more than on profits”.  **Values: beliefs-ideals: The** important and enduring beliefs, ideals, and convictions shared by the members of a culture about what is good or desirable and what is not. Values exert major influence on individual behavior and serve as broad guidelines in all situations [56]. Values are the operating philosophies or principles that guide the internal conduct of the clinic as well as its relationships with its patient, staff, and partners.  Example: One “value” may incorporate the assumption that the patient is an individual and that an individualized holistic treatment approach is most appropriate.  **Norms = informal rules:** Norms are notions of right action binding upon the members of a group, and they serve to guide, control, or regulate proper and acceptable behavior. Norms are informal guidelines about what is considered normal social behavior in a service or in the clinic. In short, "The way we do things around here" [56].  Example: “We control the quality of our care by clearly defined treatment pathways”.  **Corporate identity**: The combinations of color schemes, designs, words, etc. that a firm employs to make a visual statement about itself and to communicate its business philosophy. Identity is an enduring symbol of how a firm views itself, how it wishes to be viewed by others, and how others recognize and remember it. Unlike corporate image (which is an “in there” changeable mental impression), corporate identity is “out there”—a sensory experience conveyed by such things as buildings, decor, logo, name, slogan, stationery, uniforms; it is largely unaffected by a company’s financial up and downs. Corporate identity is more or less permanent unless it is changed deliberately [56].  Example: Apple’s logographic apple; UPS’s use of the color brown  **Professional team**  By virtue of having completed a required course of studies and/or practice, a professional team is a group of persons who are formally certified by a professional body. The team’s competence can usually be measured against an established set of standards [56].  Example: Physicians, complementary medicine practitioners.  **Attitude**: Manner, disposition, feelings, position, etc., with regard to a colleague, the job or the clinic [57].  Example: Seeing oneself as a team member, competitor or supervisor.  **Communication**: The structure, content, and ways of sharing information within the team in the service and in the clinic. To facilitate communication within their services, employees will usually need to have or develop considerable interpersonal skills—such as effective speaking, writing and listening. Emphasis should be placed on procedural, clinical, and administrative communication.  Example: Regular staff meetings and case conferences.  **Teamwork**: The process of working collaboratively with a group of people in order to achieve a goal. Members of the team use their individual skills, share insights with one another, and provide constructive feedback, despite any personal conflicts between individuals. Teamwork is a crucial part of a medical service because it is necessary for colleagues to work well together and to do their best in any circumstances [56].  Example: The physician and practitioner cooperate and combine their knowledge in order to heal the patient.  **Management style:** Refers to the method of leadership that a chief physician or head of department usually employs when running his/her service in order to achieve the highest degree of effectiveness and quality from employees. [56]  Example: Autocratic, paternalistic, or democratic management styles.  **Leadership**: The people who are in a position to rule, guide, or inspire others [58].  Leadership involves   1. Establishing a clear [vision](http://www.businessdictionary.com/definition/vision-statement.html), 2. Sharing that vision with others so that they will follow willingly, 3. [Providing](http://www.businessdictionary.com/definition/provider.html) the [information](http://www.businessdictionary.com/definition/information.html), [knowledge](http://www.businessdictionary.com/definition/knowledge.html) and [methods](http://www.businessdictionary.com/definition/method.html) to realize that vision, and 4. Coordinating and balancing the conflicting [interests](http://www.businessdictionary.com/definition/interest.html) of all [members](http://www.businessdictionary.com/definition/member.html) and [stakeholders](http://www.businessdictionary.com/definition/stakeholder.html). [56]   Example: Steve Jobs and, later, Tim Cook at Apple were able to create that company’s own culture and vision and to bring it to success.  **Medical expertise**: The basis of credibility of a [person](http://www.businessdictionary.com/definition/person.html) who is perceived to be knowledgeable in an area or [topic](http://www.businessdictionary.com/definition/topic.html) by virtue of his or her studies, [training](http://www.businessdictionary.com/definition/training.html), or [experience](http://www.businessdictionary.com/definition/experience.html) in the subject matter [56].  Example: Formal academic credentialing (such as a medical school diploma), skill training and practice, research activities and publications, and real-world experience may each contribute to perceptions and conceptualizations of medical expertise.  **Patient**  One who receives medical attention, care, or treatment [59].  Example: A woman with breast cancer who comes to the clinic for an operation.  **Attitudes**: The manner, disposition, feelings, positions, etc., between the patient and the professional team (physician, practitioner and nurses) [57].  Example: Seeing the patient as a client.  **Time devoted to the patient**: The period that is designated for and available to the exchange between a patient and a physician/practitioner [59].  Example: Consultations of up to 60 minutes with the complementary medicine practitioners  **Patient-physician/practitioner interaction**: All expectations, communications and behaviors between the physician/practitioner and patient [39]. Emphasis should be placed on building and strengthening relationships between and among people.  Example: A cooperative or asymmetric relationship in which the patient depends on a physician or in which the clinic is a service provider for the patient (the patient is seen as a client)  **Communication**: The exchange and sharing of thoughts, messages, feelings or information by speech, signals, writing, or behavior between the patient, physician, practitioner and nurses [59].  Example: Shared-decision making.  **Accessibility**: The availability of the physician/practitioner to the patient [59,60].  Example: Availability of the physician/practitioner by phone. |
| --- |
| **STRATEGY**  A method or plan of action chosen by the clinic manager and designed to achieve a desired future or a long-term goal or outcome [56,61].  Example: The broadest and most appropriate range of treatment with the best results for female breast cancer in Germany.  **Institutional strategy**: The overall [scope](http://www.businessdictionary.com/definition/scope.html) and direction of the clinic chosen by the clinic manager to [achieve](http://www.businessdictionary.com/definition/achieve.html) its mission, vision and particular major [goals](http://www.businessdictionary.com/definition/goal.html) in the health care system and in the hospital [56].  Example: Geographic scope of operation; patient groups served, such as the indigent.  **Alliance of entities**: The creation of a unique organizational entity achieved by combining the different medicine types [56].  Example: Creating an integrative medicine unit by combining conventional and complementary medicine with a high level of integration that exploits the “best of both worlds“.  **Medical model**: Describes the type of care that is offered (in the treatment portfolio).  Example: Services provided in conventional care (e.g., clinic naturopathy), services provided in complementary combined with conventional care (as in an integrative breast cancer center), or specialized services in addition to conventional procedures (e.g., acupuncture during chemotherapy).  **Service**: The performance and quality of staff’s work or duties [59].  Example: The type of service offered (e.g., acupuncture) and its quality.  **Growth:** An increase in size, number, value, or strength [59].  Example: The number of patients seen in the clinic has grown by 20%. |
| **ORGANIZATIONAL ELEMENTS**  A series of actions, changes, or functions that brings about a result or a known goal set by the strategy of the clinic [59].  Example: Improving documentation and transparency in order to facilitate research.  **Resources**: The people, assets, materials, or capital that can be used by the clinic to accomplish a goal [62].  Example: The deployment of resources, such as rooms, labor force, or time, must be planned to ensure effectiveness and efficiency.  **Visibility**: The degree to which a clinic’s reputation is disseminated (role, function, location, purpose) [59].  Example: Everyone in the region knows the clinic.  **Network**: A network is an extended group of acquaintances and associate clinics with similar interests or concerns that interact and remain in informal contact through regular communication for mutual assistance, benefits or support.  Networking is based on the question "How can I help?" rather than "What can I get?" [56,59].  Example: MD Anderson could “network” with the Breast Cancer Center in Essen; the facilities exchange information about their treatment successes. |
| **OUTCOME**  Determining and evaluating the results of an activity, plan, process, or program and their comparison with the intended or projected results [56].  Example: A high level of remission rates was achieved and, as anticipated, is aligned with the projected demand for hours, interventions and costs to achieve this goal.  **Productivity-Success**: A measure of the efficiency of a person or service. The comparison of what is actually produced or performed with what can be achieved with the same consumption of resources (money, time, labor, etc.). Productivity-success measures are important for determining productivity [56].  Example: The number of cases per months, number of procedures, etc.  **Research**: Systematic investigation intended to establish facts or principles or to collect information on a subject and publish it [58].  Example: Evaluating the effectiveness of acupuncture for chronic pain in a clinical trial.  **Quality**: The totality of features and characteristics of a product or service that reflects its ability to satisfy stated or implied needs (ISO 8402-1986 standard) [56]. Applied to clinics, quality is defined by the key outcomes (patient health) and how to achieve those at the lowest costs.  Example: Cancer remission rates, etc.  **Patient satisfaction**: The patient’s level of approval when comparing the perceived performance of the care service with his or her expectations [56].  Example: “In the integrative medicine clinic for my cancer, the team proposed different treatments and the team members were competent and kind.”  **Job satisfaction**: The degree of contentment stemming from employees’ positive and negative feelings toward their work [56].  Example: “Since I have worked at the integrative medicine center, I have more responsibility, and, consequently, I love my job much more!” |
