# Supplementary material for: MERGING conventional and complementary medicine in a clinic department – a theoretical model and practical recommendations
Source: BMC Complement Altern Med. 2015 Jun 9;15:172. doi: 10.1186/s12906-015-0696-2 (PMC4459674; doi:10.1186/s12906-015-0696-2)
Supplement: Additional file 2: Table S2. — Detailed steps to successfully offer integrative medicine. [file 12906_2015_696_MOESM2_ESM.doc]

**Additional file 2: Table S2: Detailed steps to successfully offer integrative medicine**

| **Courtship stage**  **Set strategy**  Research on the status of complementary and alternative medicine and conventional medicine   - **Motives**: *(Alliance of entities/Strategy)*   Explore the administration’s reasons for and interest in offering integrative oncology.   - **Merger or diversification:** (*Alliance of entities/Strategy)*   (Is the new entity a wholly integrated new service or does it simply provide “add-ons” or supplemental services?)  Clearly define the role of both sides in the new entity and make it transparent (integrating complementary medicine into conventional care in one department; integrative medicine as an added service or the development of a new concept (merger of equals: 50/50?)  Adapt your concepts to the clinic´s philosophy.   - **Medical model:** *(Strategy)*   Define the overall medical model, take aspects of credibility and safety into account and define how much specialization is reasonable and necessary to be competent and effective without losing the holistic approach. Offer a medical model that suits the needs of the clinic.   - **Services:** *(Service/Strategy)*   Define the number and type of different services to be offered. Establish the standard of quality to be provided and determine in which quantity each service will be offered (for example, offering three high-quality complementary medicine services—acupuncture, aromatherapy, phytotherapy—with a focus on acupuncture). Adapt the number of services to the available resources (e.g., how a service is offered to other departments—passive or active, dependent upon resources available).   - **Efficiency:** *(Productivity - Success/Outcome)*   Establish the metrics necessary to measure clinic productivity. Establish benchmarks for clinic goals.   - **Culture:** *(Values + Norms/Corporate philosophy)*   Explore the cultural differences and the breadth of the gaps between medical models. Establish how differences and gaps will influence the corporate culture of the new entity. Clarify the differences to everyone on the team.   - **Integration Team:** (*Resources/Organization + Corporate identity/Corporate Philosophy)*   Create an integration team that will identify the strengths of the merger and present them with confidence. The team should design and implement the new corporate culture for the clinic and choose a slogan, logo, building and type of decor that incorporate the new philosophy. Choose an integration team with strong leadership competencies.   - **Resources**: *(Resources/Organization)*   Ask the different therapists what kinds of resources are required:   - - Staff: Choose good, competent and sufficient personnel resources for the integrative medicine project.   - Rooms: Sufficient rooms must be provided for all therapists. Some rooms may require adaptation to the needs of integrative medicine therapies. - **Recruitment:** *(Management style /Professional team)*   Interview potential staff members using “what-if” scenarios that they might typically experience in the future. Candidates should respond in ways that suggest that they understand the goals and objectives of integrative medicine and of the clinic. Candidates should also express enthusiasm for the project and a willingness to be collaborative team members.   - **Evaluate the situation:** *(Institutional strategy/Strategy)*   Review decisions and strategy before making the merger official. |
| --- |
| **Legal Announcement of marriage**  **announcement of the merger**  Matters of style and content   - **Messenger:** *(Leadership + Communication/Professional team)*   Carefully select a messenger to communicate all merger-related themes to ensure consistent and reliable information flow.   - **External communication:** *(Visibility /Organization)*   Evaluate the success of efforts to ensure the visibility of the hospital. Ensure that the corporate identity (slogan, logo) has been widely disseminated.   - **Internal communication:** *(Alliance of entities/Strategy + Leadership/Professional team)*   Define and over-communicate all aspects of the merger, especially the new goals, the new structure and hierarchy, the new roles of key participants, and descriptions of the new corporate culture.  Ensure that all communications reflect the language of integrative medicine and clearly communicate the terms of the marriage. Dispel rumors, reduce uncertainty.  Confirm communications with written statements to avoid confusion or distortion.   - **Culture:** *(Leadership/Professional team)*   Effectively present the new corporate culture. Outline areas of cultural differences between conventional and integrative medicines but focus on the similarities and common ground.  Create a clear, consistent and realistic understanding of the new culture as early as possible.   - **Feedback:** *(Management style/Professional team)*   Incorporate an effective feedback mechanism (group announcement, question boxes, etc.). Provide employees with both public and anonymous opportunities to ask questions. Allocate time to respond to questions. |
| **Honeymoon period**  **acculturation stage**  Changing the culture   - **Culture:** *(Values + Norms/Corporate philosophy)*   Implement the new corporate culture. Be aware of possible conflicts and resolve them expediently.   - **Observation and informal discussion** *(Leadership + Communication/Professional team)*   The messenger should maintain high visibility. At the beginning of the integration, he or she should tour the clinic and informally discuss with employees to reduce anonymity and identify any barriers or considerations that could affect the quality of transitioning to full integration. Establish organizational trust.   - **Official meetings:** *(Communication + Teamwork/Professional team)*   Establish a detailed exchange about the patients with every therapist. Implement regular meetings, at least 1–2 times a week, with all therapists, nurses etc. of the department and with sufficient time. Create an open meeting atmosphere. Encourage staff members to express their concerns.   - **Attitude:** *(Professional team)*   Ensure that colleagues respect each other and interdisciplinarity, which must be supported by the head of the integrative medicine center.   - **Staff training:**  *(Resources/Organization)*    - Train complementary medicine practitioners, ensuring that they have a basic understanding of conventional diagnoses, laboratory parameters, etc.   - Train conventional staff, ensuring that they have a basic understanding of complementary medicine’s underlying concepts and methods. - **Team-building initiative:** *(Leadership + Teamwork/Professional team)*   Create space for informal meetings and easy communication, e.g., an employee kitchen/break room. Support break times for conversation and casual interactions among employees; offer prevention programs such as group yoga; organize team outings, excursions, etc. Organize recreational and casual activities by involving members of both medicines in a non-threatening and participative environment.   - **Employee survey:** *(Job satisfaction/Outcome)*   Anonymously assess current attitudes (also trust, commitment, job satisfaction, employee stress, etc.) and cultural fit through an employee questionnaire. Establish a pre-integration measure to provide a baseline for monitoring the progress or success of the cultural change.   - **Supervision of the team:** *(Management style + Communication/Professional team)*   Organize regular, outside, expert supervision to ensure productive conflict resolution.   - **Documentation:** *(Communication/Professional team)*   Design one, system-wide, common form of documentation for all interventions that accommodates the inclusion of both conventional and complementary treatments and information. Remind all staff members to regularly read all notes in the documentation.   - **Transparency:** *(Communication/Professional team)*   Utilize adequate and sustainable communication structures to ensure the flow of information and transparent decision-making. Provide consistent and regular feedback to the referring MDs/nurses about the conditions of their referred patients, which treatments are provided, and relevant patient progress.   - **Visibility:** *(Organization)*   Consistently offer the departments that refer patients for integrative services information about activities, treatments, clinical practices, etc. Offer open house presentations and in-services about integrative medicine therapies offered, including their evidence and safety. |
| **Establishing Marital allegiance**  **Establishment of the merger**  Visibility and warning signs   - **Communication:** *(Leadership /Professional team)*   The messenger should continue to maintain high visibility.   - **Networking:** *(Organization)*   Network with other integrative medicine clinics; explore the “lessons learned” from their experiences.   - **Proactive management style:** *(Management style/Professional team)*   Be aware of signs of potential difficulties:   - Increase in patient complaints - Low level of employee participation in clinic social events - Poor uptake of employee training - Increase in short-term absence - Poor time- and record-keeping - Increase in “personality clashes” |
